# Ginseng alleviates folliculogenesis disorders via induction of cell proliferation and downregulation of apoptotic markers in nicotine-treated mice

**DOI:** 10.1186/s13048-022-00945-x

**Published:** 2022-01-23

**Authors:** Masoumeh Faghani, Sahar Saedi, Korosh Khanaki, Fahimeh Mohammadghasemi

**Affiliations:** 1 Department of Anatomy, School of Medicine, Guilan University of Medical Sciences, Rasht, Iran; 2 Medical Biotechnology Research Center, School of Paramedicine, Guilan University of Medical Sciences, Rasht, Iran; 3 Cellular & Molecular Research Center, Department Of Anatomy, School of Medicine, Guilan University of Medical Sciences, Rasht, Iran

**Keywords:** Apoptosis, Nicotine, Ovary, Panax ginseng, Proliferation

## Abstract

**Background:**

Ginseng is a powerful phytoestrogen with high antioxidant properties.

**Objective:**

This study aimed to evaluate the effect of Panax Ginseng (PG) on folliculogenesis, proliferation, and apoptosis in the ovary impaired by nicotine.

**Methods:**

Forty adult mice were divided into five groups. Control, sham, and nicotine groups, and co-treated groups of nicotine and ginseng in doses of 0.5 and 1 g/kg. Folliculogenesis was assessed via histopathology and serum evaluation of estradiol, progesterone, follicle-stimulating hormone (FSH), and luteinizing hormone (LH) by ELISA. Lipid peroxidation and antioxidant enzyme activities both in homogenate tissue and serum were assayed by colorimetric analysis. Apoptotic markers of cytochrome c (Cyt *c*), Bax, and Bcl-2 were evaluated by RT-PCR. Proliferative index was studied by the Ki-67 immunostaining procedure.

**Results:**

In comparison to the control or sham groups, nicotine significantly reduced the levels of FSH, LH, and estradiol hormones. An insignificant reduction was observed in the progesterone hormone. Nicotine reduced all healthy follicle numbers, except primordial (*P* = 0.001). Malondialdehyde (MDA) was increased in tissue and serum in the nicotine group (*P* = 0.01). Serum catalase (CAT) and enzymatic activity of superoxide dismutase (SOD) both were reduced in tissue and the serum, in the nicotine group. Nicotine induced a reduction in the proliferative indexes of granulosa and theca cells in pre-antral and antral follicles (*P* = 0.001). However, its effect on the proliferative index of stroma cells was not significant. Apoptotic markers were elevated in the nicotine group (*P* = 0.001). Co-treatment with ginseng elevated all sex hormones, increased healthy follicles, and reduced tissue or serum lipid peroxidation, compared with the nicotine group (*p* < 0.05). Co-Treatment with ginseng also reduced the expression of apoptotic markers and increased the proliferative indexes in granulosa and theca cells in pre-antral and antral follicles and also in stroma cells, in comparison to the nicotine group (*P* = 0.001). All above-mentioned alterations following treatment with ginseng were remarkable, especially in the dose of 1 g/kg.

**Conclusion:**

This study showed ginseng protects folliculogenesis via alteration of hypothalamic- pituitary–gonadal (HPG) axis, induction of proliferation in ovarian somatic cells, reduction of lipid peroxidation, and downregulation of apoptotic markers in the mouse ovary, treated with nicotine.

## Introduction

Nicotine, as one of the important ingredients of cigarettes, disturbs the function of the cardiovascular, hepatic, urogenital, nervous, and endocrine systems [1, 2]. Besides, researchers have shown that nicotine causes fertility disorders in both genders [3, 4]. Physiology of endocrine glands of pituitary, thyroid, adrenal, testis and ovary altered following nicotine treatment [2, 4, 5]. Changes in sex hormones following the impact of nicotine appear to affect the release of hypothalamic-releasing hormones through hypothalamic-pituitary–gonadal (HPG) axes, such as luteinizing hormone-releasing hormone (LHRH) [2].

Following treatment with nicotine, follicle-stimulating hormone (FSH) increases. FSH levels in women smokers are higher than non-smokers [6]. Nicotine affects the morphology of the female reproductive tract in animal studies [5, 7]. It not only disrupts the luteal phase by inhibiting progesterone secretion [8], but also causes morphological changes in the endometrium and epithelium of the fallopian tubules, and reduces the number of glands and mucosal folds [5].

In vivo studies of mice treated with nicotine have shown reduced numbers of pre-antral and antral follicles, corpus luteum, and estradiol reduction [8]. In vivo study in treated rats by nicotine also resulted in the reduction of follicle numbers, and ovarian cholesterol and glycogen elevation [9]. Inhalational cigarette smoking in mice has adverse effects on oocyte quality as it increases zona pellucida thickness and alters the metaphase II spindle formation [10].

Nicotine may increase cell stress and apoptosis rate in reproductive system [11]. In this regard, researchers have shown that smoking in men and women increases apoptosis in the urogenital system [2, 12]. Apoptosis of granulosa cells in rat ovarian follicles increases, following treatment with nicotine [13]. Nicotine elevates reactive oxygen species (ROS), resulting in cell and tissue damages and also apoptosis [14]. ROS have different effects on the physiological function of the female reproductive system including, folliculogenesis, oocyte maturation, ovulation, corpus luteum shape, endometrial cycle, implantation, and pregnancy [15].

In vitro studies on the oocyte have shown that nicotine blocks meiotic division in metaphase-I, through meiotic spindle formation and chromosome disturbances [16, 17]. Nicotine reduces cell number and disrupts cell diploidy in the bovine blastocyst, and disturbs embryonic development [18]. The proliferation of granulosa cells in rat ovaries is reduced by nicotine treatment [13]. Fetal or neonatal exposure to nicotine also reduces proliferation and elevates apoptosis of granulosa cells in rats [19].

As the number of women who smoke in modern societies has increased [20], it is necessary to find alternative methods to reduce the harmful effects of smoking and especially nicotine.

Phytoestrogens are dietary compounds found in various types of foods. Ginseng is a powerful phytoestrogen with high antioxidant properties. There are various reports about the beneficial effects of ginseng on various disorders of the systems such as cardiovascular, endocrine, immune system, menopausal symptoms, and reproduction [21, 22].

Ginseng can modulate cellular activity and function through genomic and non-genomic signaling. It binds to the cell surface and nuclear receptors such as estrogen receptor (ER), progesterone receptor, androgen receptor, and proliferator-activated receptor [23]. Structurally, ginseng has similarity to 17-β-estradiol (E2), in binding to the ER [24].

Ginseng increases the maturation of mouse pre-antral follicles and the production of steroids and proliferating cell nuclear antigen (PCNA) [23]. Moreover, it has anti-aging, antioxidant, anti-tumor, and anti-stress properties [25, 26]. It has been shown that ginseng has significant estrogenic activity, and can regulate the estrous cycle [27].

Research has shown that ginseng extract, due to its high antioxidant properties increases the number of ovarian follicles, and subsequently increases sex hormones, and reduces the number of antral follicles [28]. Ginsenoside compound K derived from ginseng has anti-apoptotic, anti-inflammatory, and anti-oxidative effects; it also protects porcine oocyte meiotic maturation against damages induced by benzo(a)pyrene [29].

The process of aging of the ovary and premature ovarian failure in rats is prevented by ginseng [30]. The number of germ cells and PCNA expression as a proliferation marker increases in the chicken’s ovary, following treatment with ginseng [18]. Additionally, previous in vitro studies have reported the pro-proliferation effects of ginseng on various cells, such as oocytes, neurons, and endothelial cells [18, 31]. In this study, we aimed to investigate the effect of Panax Ginseng (PG) on folliculogenesis, apoptosis, proliferation index, and oxidative status in the mouse ovary, impaired by nicotine.

## Materials & Methods

Forty adult female NMRI mice with a mean weight of 35 ± 5 g, and an age of 8 − 10 weeks were selected. Animals were maintained for one week in the animal house in their cages for acclimation. All animals were in groups of four in mice cages; they were maintained in standard condition: The light–dark cycle of 12 h, 23 − 25 °C, and humidity.

Animals were randomly divided into five groups (each group, *n* = 8) as follows, A: Control group, received no drug, B: Sham group, received 1% carboxymethyl cellulose (CMC)-saline, (Sigma-USA) orally, C: Nicotine group, received nicotine (Sigma-USA), 0.6 mg/kg intraperitoneally, D: Co-treated group, received nicotine 0.6 mg/kg intraperitoneally, and PG in the dose of 0.5 g/kg and E: Co-treated group received 0.6 mg/kg nicotine intraperitoneally, and PG in the dose of 1 g/kg. PG was administered through gastric gavage by gauge 20. All treatments were performed once a day and lasted for 30 days.

PG powder was received as a gift from the Barij Essence Pharmaceutical Co. Nicotine was diluted with normal saline, and PG was dissolved in 1% CMC-saline before treatment. The selected dosage of nicotine approximately was equivalent to whole human subjects, who smoke 12 cigarettes per day [32]. The dosage of PG was chosen on the bases of previous studies [33].

### Determination of the estrous cycle

At the beginning of the study, all animals were selected and treated when they were in their estrous phase of the estrous cycle. Animals also were checked for the estrous phase of the estrous cycle at the end of the experiment.

The estrous cycle of all animals was checked at 8 − 9 am by vaginal cytology assessment. Vaginal smears were prepared on a slide. After drying, they were stained with Papanicolaou (PAP) stain.

The estrous cycle has four stages, namely proestrus, estrous, metestrus, and diestrus. Proestrus stages are characterized by the presence of a high ratio of nucleated epithelial cells and a few cornified epithelial cells. The estrous stage is also characterized by a high ratio of cornified eosinophilic cells. The metestrus phase is a short stage, which is characterized by a large number of leukocytes, and a few cornified epithelial cells. In diestrus as a long stage, mainly leukocytes are observed [34].

### Animal surgery

At the end of the experiment, all animals were anesthetized with an intraperitoneal (i.p.) injection of ketamine and xylazine. Blood samples were obtained from inferior vena cava for biochemical assays. Both ovaries were removed. The left ovary was put in 10% neutral buffered-formalin for fixation and histological studies. The right ovary was used for molecular evaluations by real-time-PCR (RT-PCR), and evaluation of tissue MDA and enzymatic activity of SOD.

### Biochemical assays

Blood was centrifuged for 10 min at 4000 rpm. The serum was separated and stored at -80 °C until further analysis by the biochemical assay.

### Hormone analysis

Serum estradiol and progesterone were measured using an ELISA kit (Monobind –USA) with a sensitivity of 8.2 pg/mL and 0.1 ng/mL, respectively. FSH and luteinizing hormone (LH) were measured, using an ELISA kit (Abnova- USA). The test sensitivity for FSH and LH were also 0.5 mIU/mL and 0.1 mIU/mL, respectively. All protocols for hormone assay were done based on the kit’s instructions.

### Serum lipid peroxidation and antioxidant status

Malondialdehyde (MDA) activity in serum was measured for lipid peroxidation study using colorimetric methods (Zellbio, Germany) under the manufacturer’s recommendations. For the study of serum antioxidant status, CAT and SOD activities were also assessed by colorimetric methods (Zellbio, Germany). Detection limit for CAT, MDA, and SOD were 0.5 U/mL, 1 U/mL, and 0.1 µm, respectively.

### Preparation of ovary homogenate

In brief, the tissues were weighed and homogenized in 1 ml ice-cold phosphate-buffered saline (PBS), then centrifuged at 14,000 × g for 5 min at 4 °C. The resulting supernatants were collected; it was kept at -80 °C, until further analysis; MDA was determined by a colorimetric assay kit (Teb Pazhohan Razi (TRP), Iran) according to the manufacturer’s guidelines. Briefly, MDA and thiobarbituric acid (TBA) formed a complex, and then the absorbance was recorded at the wavelength of 545 nm. The results were expressed as µmol/mg protein.

The SOD activity of supernatants was assessed through a commercial kit (Teb Pazhohan Razi (TRP), Iran), which was based on a colorimetric method (450 nm). It should be noted that an enzyme reaction occurred in this assay, in which superoxide anion is transformed to hydrogen peroxide and oxygen. The data were reported as a unit (U)/mg protein. The total protein content of the supernatants was measured using the Bradford method [35].

### Histology and follicle count

Fixed ovary tissues were applied for routine tissue preparation and embedded in paraffin. In the next step, the tissues were sectioned with a microtome (Leitz, Germany) at 5 µm thickness and stained with hematoxylin and eosin (H&E). Folliculogenesis was studied by counting the number of various follicles, including primordial, primary, pre-antral, antral, corpus luteum, and atretic follicles.

Follicles were categorized based on their morphology. Primordial follicles were the smallest ones. They were characterized by an oocyte and one layer of squamous follicular cells. Primary follicles were larger than primordials, characterizing with an oocyte and one layer of cuboid follicular cells. Pre-antral follicles had 2 − 8 or more layers of granulosa cells without an antral cavity. Antral follicles have one or more small antral cavities (early antral), or one large antral cavity (late antral); in the present study, both were considered as antral follicles [36]. Atretic follicles were characterized with at least two or more criteria, including granulosa cells with a pyknotic nucleus, separation of granulosa layer from the basal membrane, degenerated zona pellucida, and oocyte fragmentation [13]. Corpora lutea were also counted. For histological examination, follicle counts were done on every 3^rd^ section of the ovary, in a way that each section was separated by a distance of approximately 15 μm from the next section used for enumeration [13]. Then, 5 slides of the total sections were selected randomly and observed with an Olympus light microscope. To avoid double-counting just follicles with oocyte-containing nuclei were counted [37, 38].

### Detection of Bax, Bcl-2, and (Cyt *c*) expression in the ovary using RT-PCR.

The ovary tissue pieces were immediately transferred to liquid nitrogen, until further analysis. Total RNA was extracted using the Sinaclone kit (Iran) according to the company’s guidelines. The purity of the extracted RNA was evaluated by measuring the absorbance at 260/280 nm, using the Nanodrop spectrophotometer (Thermo Fisher Scientific Inc., USA). Then, the extracted RNA was treated with DNase to eliminate possible contamination. cDNA was synthesized using a cDNA synthesis kit (BioFACTTM, South Korea). Relative mRNA expression of Bax, Bcl-2, and cytochrome c (Cyt c) was evaluated using RT-PCR with the ABI instrument (StepOneTM, USA). The glyceraldehyde 3-phosphate dehydrogenase (GAPDH) gene was considered as a reference gene for gene expression normalization.

PCR Primers for the above-mentioned genes were designed with Primer3web (version 4.0.0). The designed PCR primers were confirmed using the Primer-BLAST system, available at the National Center for Biotechnology Information (NCBI). PCR primer sequences were summarized in Table 1. All reactions were run in triplicate (*n* = 3).

**Table 1:** Primer sequences used for quantitative Real-Time PCR

**Genes**	**Primer sequences (5′ 3')**	**Optimized annealing** **Temperature(C°)**	**Product Size** **(bp)**
**Bax**	**F:** GCTGCAGACATGCTGTGGATC **R:** TCACAGCCAGGAGAATCGCAC	60	419
**Bcl-2**	**F:** TTAGAGAGATGCGAGGAACCG **R:** GGGACAAGTAAACCTGGAAGAA	60	158
**Cyt c**	**F:** CGGCTGCTGTGATTGTGAAT **R:** TGTCTTGTGTTTCCCGCCTT	60	157
**GAPDH**	**F:** AACTTTGGCATTGTGGAAGG **R:** ACACATTGGGGGTAGGAACA	60	223

### Proliferative index of Ki-67 immunohistochemistry

Proliferative index in follicles and stroma were done using immunohistochemistry and Ki-67 marker. Tissues were sectioned on poly L-lysine coated slides with 3 µm thickness. Sections were deparaffinized and rehydrated. Antigen retrieval was done in EDTA-Tris buffer (pH = 9), inside a microwave at 120 °C for 40 min. The endogen peroxidase activity was inhibited, by 3% hydrogen peroxide (H_2_O_2_). After washing with PBS, bovine serum albumin (BSA) was used for inhibition of non-specific antibodies binding. The sections were incubated with a ready-to-use rabbit monoclonal anti-mouse Ki-67 antibody (Zytomed-Germany) for 80 min in a wet chamber at room temperature. After washing, a mouse rabbit enhancer (Bioassay-USA) was used for 30 min for label attaching.

After washing, the mouse-rabbit horseradish peroxidase (HRP), a label (Bioassay-USA) was used for 30 min at room temperature. Sections were washed with PBS three times for 5 min. Afterward, diaminobenzidine (DAB) (Bioassay-USA) was applied as a chromogen for 5 min. Subsequently, sections were counterstained with Mayer’s hematoxylin and prepared for observation by a light microscope.

Two slides of each animal were stained. The brown cells were considered as Ki-67 positive cells. Digimizer image analysis software version 5.7.0 was used for counting the numbers of proliferative cells in both follicles and stroma in the same area.

The proliferation indexes of granulosa, theca, and stroma cells were calculated by the numbers of Ki-67 positive cells in proportion to all cells. Then, the answer was multiplied by 100 and expressed as a percentage [13, 39].

### Statistical analysis

For statistical analysis, SPSS software version 26 and Graph Pad Prism version 7 were used. At first, using Kolmogorov–Smirnov (KS) test, the normality of data distribution was tested. Analysis of variance (ANOVA) test followed by post hoc Tukey test was used for data comparison among groups. In the case that the data were not normally distributed or in the lack of homogeneity of variance assumptions, the Kruskal–Wallis non-parametric test was performed. Data were reported as the mean and standard error (Mean ± SEM). The value of *P* < 0.05 was considered significant.

## Results

### Hormones

**Fig. 1:**
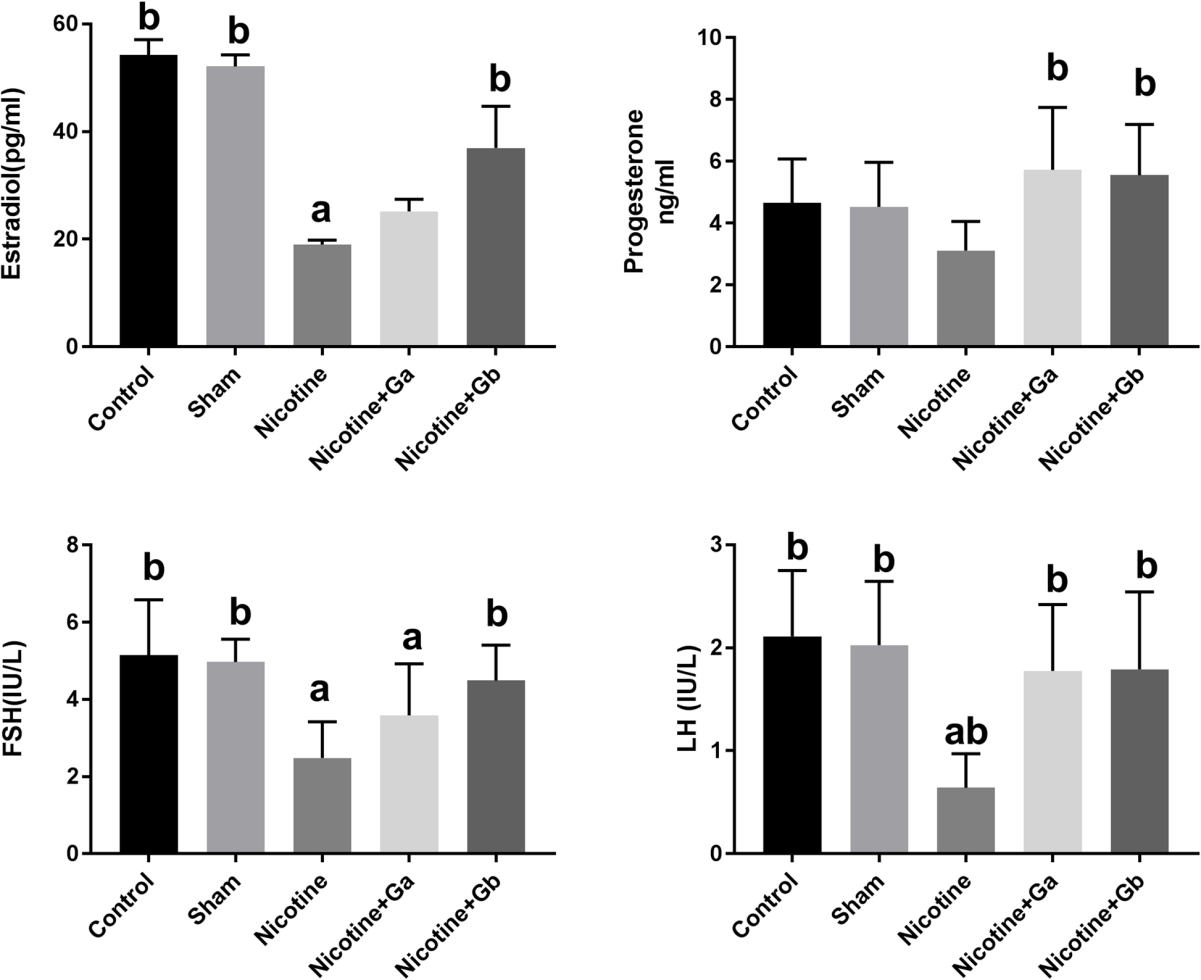
Serum concentration of estradiol, progesterone, FSH, and LH in mice in different groups. Data are expressed as Mean ± SE. There are 8 mice in each group (*n* = 8). a: Significant data in comparison to the control (*p* < 0.05), b: Significant data compared to the nicotine-treated group (*p* < 0.05)

Administration of nicotine significantly reduced the serum estradiol level, compared to the control group or sham (*p* = 0.03). Co-administration of PG and nicotine increased the level of serum estradiol, compared to the nicotine group; and it was significant in the 1 g/kg PG-treated group (*p* = 0.04) (Fig. 1). Progesterone level was lower in the nicotine group, compared to the control and sham groups; however, it was not significant (Fig. 1). In co-treated groups with nicotine plus 0.5 or 1 g/kg ginseng, the level of progesterone increased, in comparison to the nicotine group (*P* = 0.01).

The level of FSH and LH were significantly lower in the nicotine group, in comparison to the control and sham (*P* = 0.001). Treatment with nicotine plus ginseng increased the level of FSH, compared to the nicotine group; and it was significant (*P* = 0.001) in the nicotine plus 1 g/kg ginseng group (Fig. 1). The level of LH significantly increased in co-treated groups, in comparison to the nicotine group (*P* = 0.001) (Fig. 1).

### Oxidative marker and antioxidant enzymes

**Fig. 2:**
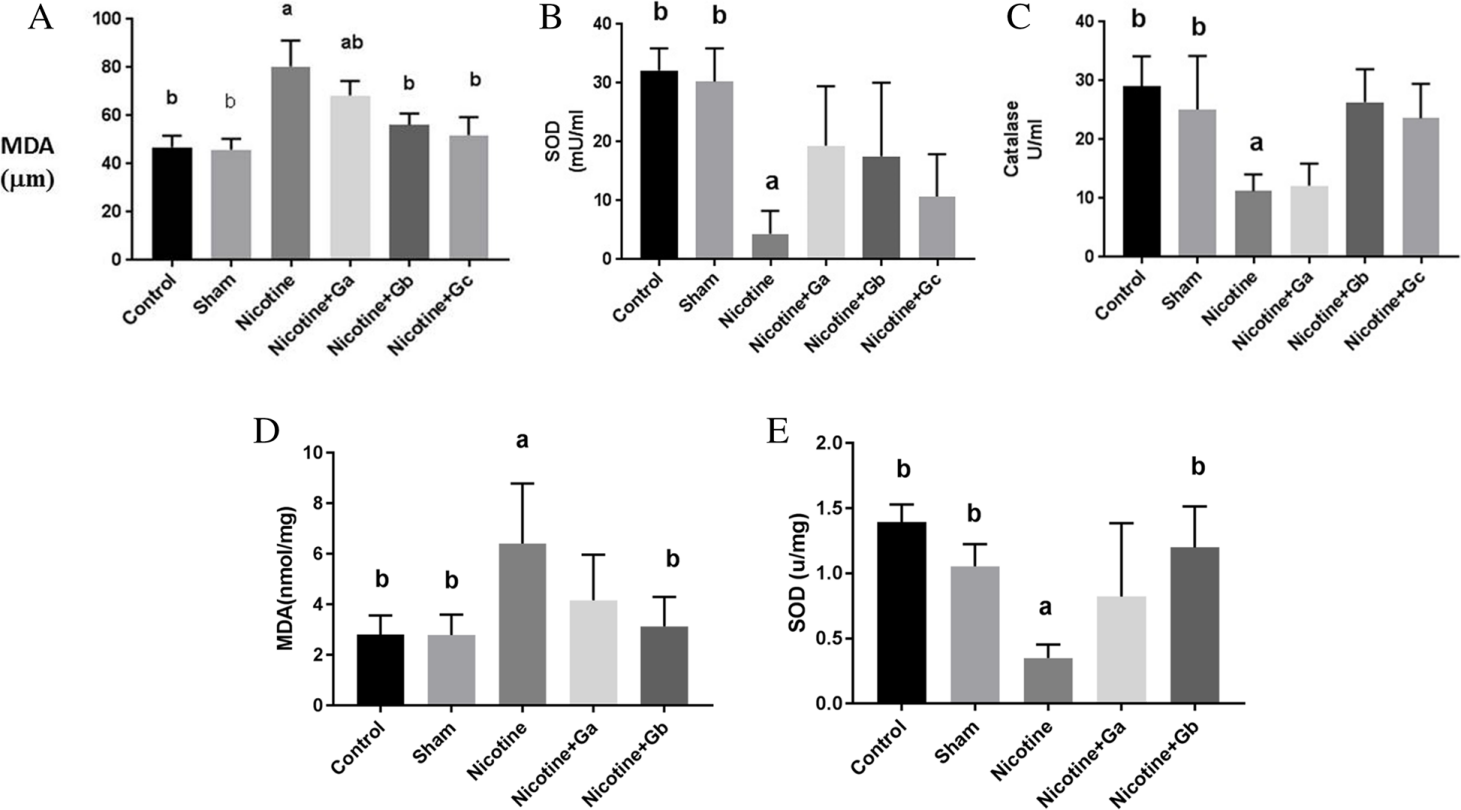
**A**, **B**, and **C** show lipid peroxidation and antioxidant markers in serum. **D** and **E** show lipid peroxidation and antioxidant markers in ovary tissue homogenate in different groups. Data are expressed as Mean ± SE. Each group has 8 mice (*n* = 8). a: Significant data in comparison to the control (*p* = 0.01), b: Significant data compared to the nicotine-treated group (*p* = 0.001)

Nicotine caused a significant rise in serum MDA level as an indicator of lipid peroxidation, in comparison to the control or sham groups (*P* = 0.01) (Fig. 2). Co-treated groups with nicotine plus ginseng (0.5 or 1 g/kg) exhibited a decrease in serum MDA level, compared to the nicotine group (*P* = 0.001). Both serum SOD and CAT activities were lower in nicotine-treated groups, in comparison to the control or sham groups (*P* = 0.001). Co-treatment with nicotine plus ginseng (0.5 or 1 g/kg) elevated the serum CAT activity, in comparison to the control or sham groups; and it was significant in the group treated with 1 g/kg ginseng (*P* = 0.001). An insignificant increase was observed in serum SOD level in ginseng-nicotine-treated groups, compared to the nicotine group (Fig. 2). Tissue MDA in the nicotine group was higher than control and sham groups (*P* = 0.01) (Fig. 2). Treatment with nicotine plus ginseng in the dose of 1 g/kg reduced the tissue MDA level, in comparison to the nicotine group (*P* = 0.001). A lower tissue SOD level was observed in the nicotine group, in comparison to the control and sham groups (*P* = 0.01). Treatment with nicotine plus ginseng in the dose of 1 g/kg could increase the tissue SOD level, in comparison to the nicotine group (*P* = 0.001) (Fig. 2).

### Ovarian histological findings and folliculogenesis

Ovary in controls and shams were covered by a simple cuboid epithelium. In the cortex, there were a lot of follicles in various stages; in the medulla, many blood and lymph vessels were found. All types of follicles were observed in the ovary. The architecture of the ovary in the sham group was similar to controls.

**Fig. 3:**
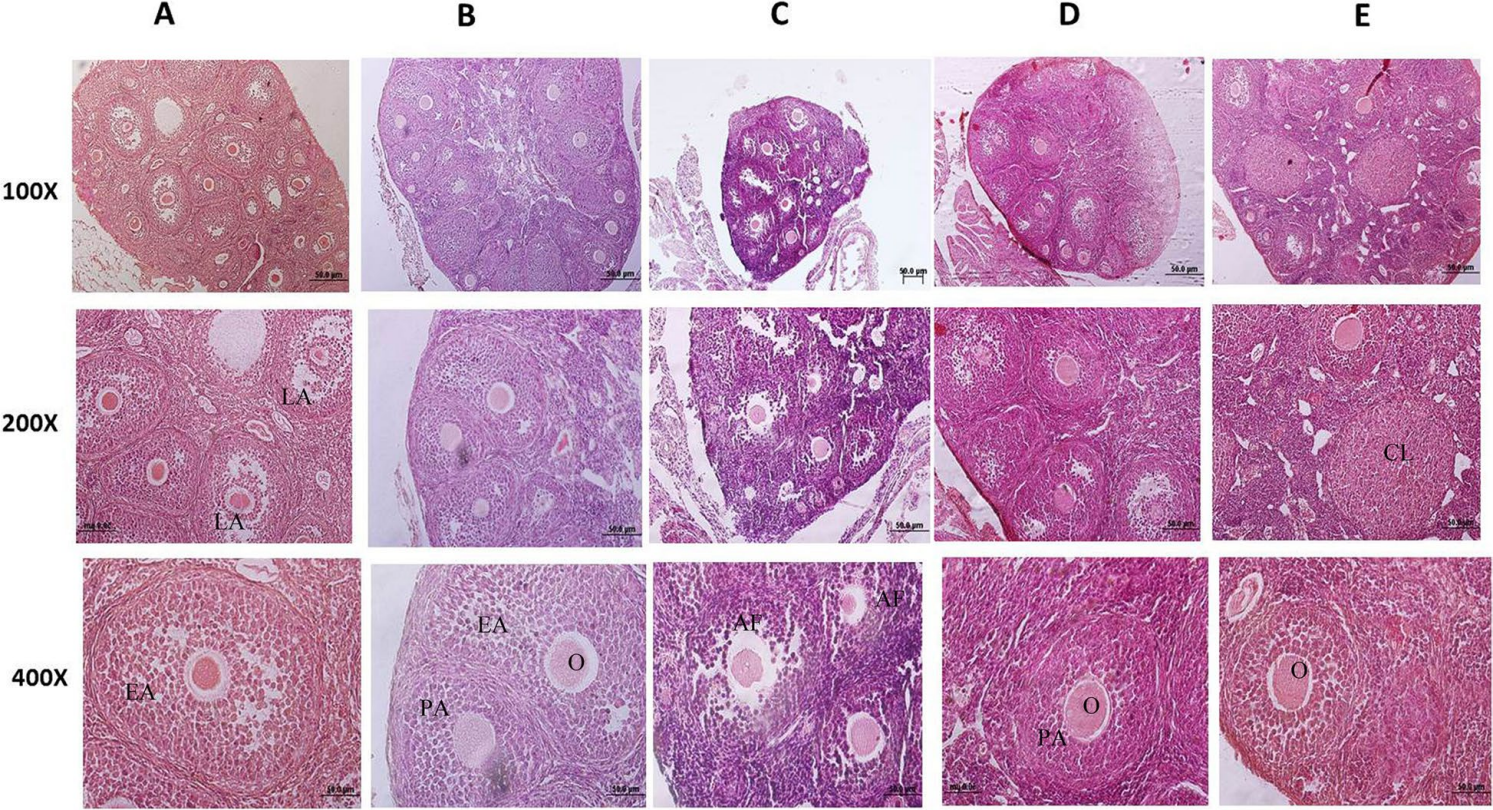
Photomicrograph of mouse ovary. Each column is showing a separate group. A: control, B: sham, C: nicotine, D: nicotine + 0.5 g/kg ginseng and E: nicotine + 1 g/kg ginseng. Each group has 8 mice (*n* = 8). PA: pre-antral follicle, EA: early antral follicle, LA: late antral follicle, O: oocyte, CL: Corpus luteum, AF: Atretic follicle. In C or the nicotine group, the reduced size of the ovary and elevation of atretic follicles are noted. Treatment with ginseng has improved ovary histology. Hematoxylin & Eosin (100 × , 200 × , and 400 ×)

In the nicotine-treated group, the ovary was atrophic (Fig. 3). Various follicles were observed, but the numbers of follicles reduced. Nicotine reduced the numbers of primary, pre-antral, and antral follicles (*P* = 0.001), compared to the control or sham groups (Table 2). However, the primordial follicles were not affected significantly by nicotine. Therefore, the numbers of total healthy follicles were reduced in the nicotine-treated group (Table 2). Follicular degeneration and morphological changes of zona pellucida were observed in most types of follicles in the nicotine group. Atretic follicles were higher in the nicotine group, in comparison to the controls and sham groups (*P* = 0.001).

**Table 2:** Effect of co-administration of nicotine and ginseng on estradiol and folliculogenesis

**Groups**	**Primordial** **Follicle (n/f)**	**Primary follicle** **(n/f)**	**Preantral follicle** (n/f)	**Antral follicle** **(n/f)**	**Corpus luteum** **(n/f)**	**Healthy follicle** **(n/f)**	**Atretic follicle** **(n/f)**
**Control**	11.14 ± 2.35	8.98 ± 0.32^b^	19.42 ± 0.50^b^	9.47 ± 0.32^b^	3.6 ± 0.35^b^	52.9 ± 0.79^b^	4.5 ± 0.54^b^
**Sham**	9.88 ± 1.32	8.99 ± 0.17^b^	18.76 ± 0.40^b^	9.29 ± 1.07^b^	3.06 ± 0.40^b^	51.7 ± 0.48^b^	4.9 ± 0.24^b^
**Nicotine**	7.43 ± 2.92	4.76 ± 0.17^a^	8.59 ± 0/95^a^	4.76 ± 0.89^a^	1.50 ± 0.22^a^	26.9 ± 0.56^a^	11/26 ± 0.89^a^
**Nicotine + Ga**	11.12 ± 1.71	7.64 ± 0.28^ab^	13.1 ± 0.71^ab^	8.02 ± 0.32^b^	2.46 ± 0.17	40.34 ± 0.71^b^	6.72 ± 0.45^b^
**Nicotine + Gb**	21.50 ± 5.18	8.26 ± 0.22^b^	15.53 ± 0.89^ab^	9.3 ± 0.32^b^	3.1 ± 0.14^b^	44.06 ± 0.7^b^	6.87 ± 0.30^b^

Data are expressed as mean and standard error. (n/f): number /microscopic field. Ga: ginseng 0.5 g/kg, Gb: ginseng 1 g/kg. ^a^Significant data in compare with control: *P* = 0.001, ^b^Significant data in compare with nicotine *P* = 0.001

Co-treatment with nicotine plus ginseng increased the total healthy follicle counts; it was significant in the 1 g/kg ginseng-treated group. The numbers of primordial follicles were not significantly different between different groups (Table 2), (Fig. 3). Also, co-treatment with nicotine plus ginseng (0.5 or 1 g/kg) significantly reduced the numbers of atretic follicles, in comparison to the nicotine group (*P* = 0.001), (Table 2). Generally, the morphology of follicles, oocytes, and zona pellucida in co-treated groups was better, in comparison to the nicotine group.

### Corpus luteum

Corpus luteum, as a marker of ovulation, was observed in all groups and it means that ovulation occurred in all groups. However, in the nicotine-treated groups, their numbers were reduced, in comparison to the controls and sham groups (*P* = 0.001). The numbers of corpus luteum were higher in the co-treated groups and it was significant in the nicotine plus 1 g/kg ginseng, in comparison to the nicotine group (*P* = 0.001) (Table 2).

### Apoptotic markers

The expression of Bax as a pro-apoptotic molecule in the ovarian tissue and the nicotine group was significantly increased, in comparison to the control or sham groups. Co-treatment with nicotine and PG in the dose of 0.5 or 1 g/kg reduced the Bax expression (*P* = 0.03), in comparison to the nicotine group. Bcl-2, as an anti-apoptotic marker reduced in the nicotine-treated group; however, it was not significant. Bax/Bcl-2 ratio in the nicotine group was higher than in other groups. Co-treatment with nicotine and ginseng in the dose of 1 g/kg significantly increased the Bcl-2 expression (*P* = 0.03), and co-treatment with nicotine plus ginseng in both doses reduced the Bax/Bcl-2 ratio, as compared to the nicotine group. Nicotine also increased the expression of Cyt *c* as an apoptotic marker (*P* = 0.01). Co-treatment with ginseng-nicotine reduced this marker, and the reduction was significant in the nicotine plus ginseng-treated groups (*P* = 0.03) (Table 3).

**Table 3:** Effect of co-administration of nicotine and ginseng on apoptotic genes in mouse ovary

**Groups**	**Bax**	**Bcl-2**	**Cyt c**	**Bax/Bcl-2**
**Control**	2.62 ± 0.2^b^	4.82 ± 2.60	2.47 ± 0.78^b^	0.53 ± 0.23^b^
**Sham**	0.83 ± 0.33^b^	3.72 ± 1.93	2.27 ± 1.46^b^	0.26 ± 0.14^b^
**Nicotine**	5.88 ± 1.16^a^	2.44 ± 1.02	19.07 ± 5.54^a^	1.60 ± 0.19^a^
**Nicotine + Ga**	1.39 ± 0.52^b^	10.22 ± 1.26	4.75 ± 2.87^b^	0.13 ± 0.04^b^
**Nicotine + Gb**	1.25 ± 0.89^b^	16.85 ± 4.83^ab^	3.34 ± 1.75^b^	0.08 ± 0.05^b^

Data are expressed as Mean ± S.E.; Ga: ginseng 0.5 gr/kg, Gb: ginseng 1 gr/kg. ^a^significant in compare to controls: *P* = 0.01; ^b^significant in compare to nicotine *P* = 0.03

### Proliferative index

**Fig. 4:**
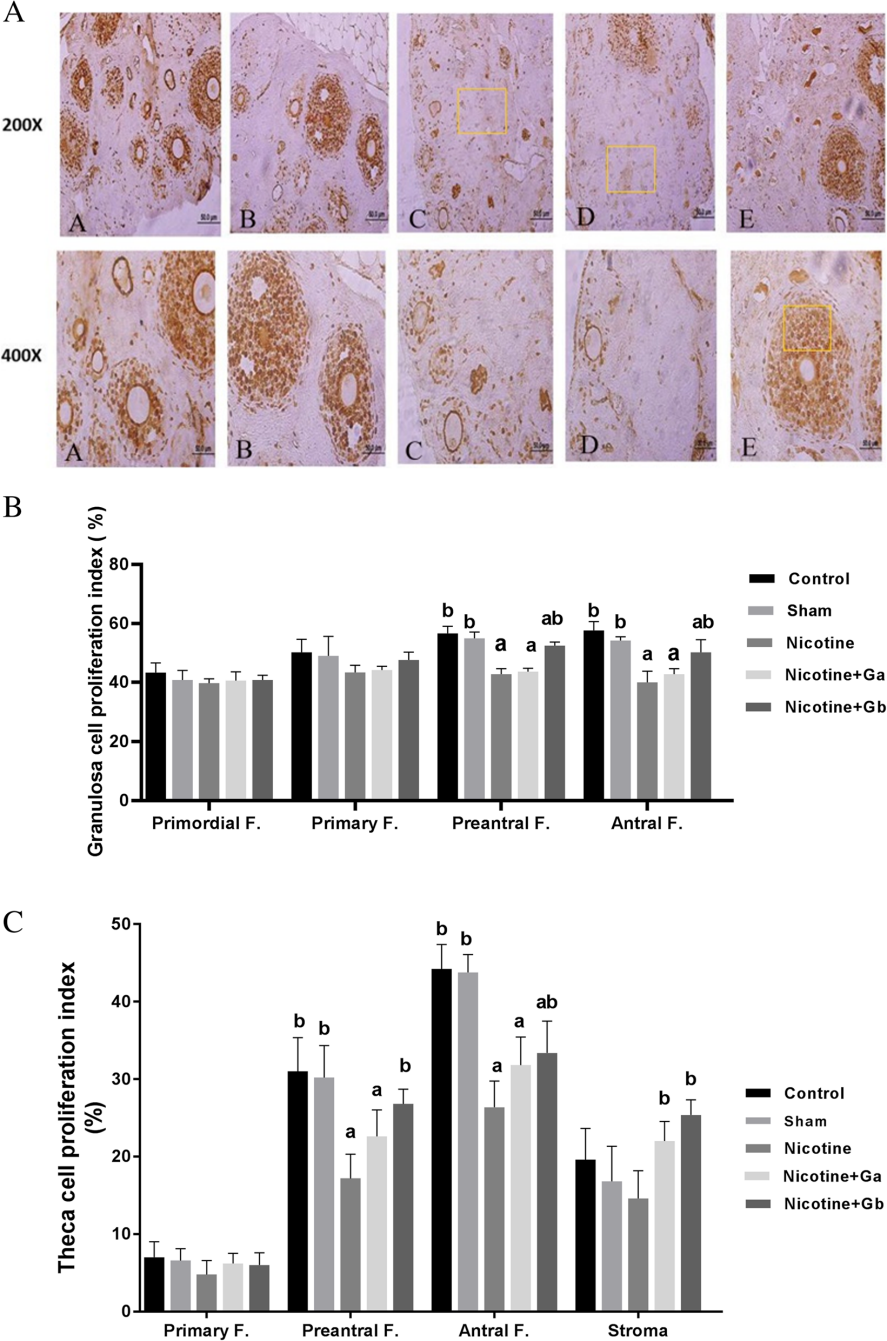
**A** Shows Ki-67 immunostaining in ovary tissue. A: control, B: sham, C: nicotine, D: nicotine + 0.5 g/kg ginseng and E: nicotine + 1 g/kg ginseng. In C or nicotine treated mouse a weak to moderate immunostaining reaction in granulosa cells is noted, in comparison to the control or sham. In E, moderate to strong immunostaining reaction and elevation of the proliferative index, are noted, in comparison to the nicotine group (200 × and 400 ×). **B** Shows granulosa proliferative index in the ovary in different groups. Each group has 8 mice (*n* = 8). Data are expressed as Mean ± SE. a: significant data in comparison with the control (*P* = 0.001), b: significant data in comparison with the nicotine group (*P* = 0.001). **C** Shows theca/stroma proliferative index in the ovary in different groups. Each group has 8 mice (*n* = 8). Data are expressed as Mean ± SE. a: significant data in comparison with the control group (*P* = 0.001), b: significant data in comparison with the nicotine group (*P* = 0.001).

Immunohistochemistry using Ki-67 nuclear antibody was used to study the proliferative index of granulosa and theca cells in various types of follicles in the ovary. The numbers of proliferative cells in the stroma also were measured in all groups. Granulosa cells mostly reacted as moderate to strong with Ki-67 immunostaining in the control and sham groups, in comparison to the nicotine group (Fig. 4). In the nicotine group, granulosa cells usually reacted as weak to moderate. The proliferative index of granulosa cells in the nicotine group in most of the follicles was significantly reduced, in comparison to the control or sham groups (Fig. 4). However, there were no significant statistical differences between different groups in the proliferative index of granulosa cells in primordial or primary follicles (Fig. 4). Lower levels of proliferative index in granulosa cells were observed in pre-antral and antral follicles in the nicotine group, in comparison to the control or sham groups (*P* = 0.001), respectively.

Co-treatment with nicotine plus ginseng in the dose of 0.5 g/kg induced a weak to moderate. Ki-67 immunostaining in granulosa cells, in comparison to the control or sham group. There was no significant difference in the proliferative index of granulosa cells in pre-antral or antral follicles between the nicotine group and co-treated group in the dose of 0.5 g/kg (Fig. 4). Co-treatment with nicotine plus ginseng in the dose of 1 g/kg induced a moderate to strong Ki-67 immunostaining in granulosa cells. The proliferative index of granulosa cells in pre-antral and antral follicles in the nicotine plus 1 g/kg ginseng-treated group increased significantly, in comparison to the nicotine group (*P* = 0.001).

The proliferative index of theca cells with Ki-67 immunostaining is shown in Fig. 4. Due to the lack of theca cells in primordial follicles, there was no Ki-67 positive cell. Generally, the proliferative index in theca cells was lower than granulosa cells in each type of follicles. There was a moderate to strong Ki-67 immunostaining in theca cells in the controls and sham groups, in comparison to the nicotine groups. No significant statistical differences were observed in theca cell proliferative index in primary follicles among groups. Nicotine reduced theca cell proliferative index in both pre-antral and antral follicles, in comparison to the control or sham groups (*P* = 0.001). A slight increase was observed in theca cell proliferative index in nicotine plus ginseng in the dose of 0.5 g/kg, in comparison to the nicotine group, but it was not significant. Co-treatment with nicotine plus ginseng in the dose of 1 g/kg induced a moderate to strong Ki-67 immunostaining in theca cells. Theca cell proliferative indexes were higher in pre-antral and antral follicles in nicotine plus 1 g/kg ginseng, in comparison to the nicotine group (*p* = 0.001).

The results related to the proliferative index in the stroma in the same area of various groups are shown in Fig. 4. A moderate to strong Ki-67 immunostaining was observed in the stroma. There were not significant statistical differences in the numbers of proliferative cells between nicotine, in comparison to the control or sham groups. Co-treatment with nicotine plus ginseng in both doses of 0.5 and 1 g/kg increased the proliferative index (*P* = 0.001), in comparison to the nicotine group (Fig. 4).

## Discussion

This study indicated the efficacy of PG, especially in the dose of 1 g/kg against apoptotic and oxidative damages of the ovary induced by nicotine. Fertility duration and women's health status depend on the reserves of ovarian follicles [40]. Follicle loss may be due to some mechanisms such as hormonal disturbances, ROS elevation, reduction of enzymatic and non-enzymatic antioxidants, the elevation of apoptotic markers, and proliferation index reduction. All of the above-mentioned factors may lead to fertility problems in women.

Estrogen is a key hormone in both the development and normal physiology of the female reproductive tract. The granulosa and interna theca cells synthesize estradiol in pre-ovulatory follicles, following stimulating by FSH. Estradiol is necessary for LH receptor expression, formation of antrum and gap junctions, and also follicular atresia prevention. It is also necessary for the proliferative phase in the uterus [41]. When estrogen reaches a critical level during oocyte maturation in the ovary, it begins to exert positive feedback on LH production, which results in the LH surge; which is essential for ovulation induction [42, 43].

Following LH surge, proteolytic enzyme activity inside the follicle increases; the ovarian wall becomes weak and produces a passage for oocyte during the ovulation process [42]. Therefore, any disruptions in the hormonal communication between FSH and LH, estrogen and progesterone, and their receptors may result in anovulation or amenorrhea, leading to fertility problems [42, 43].

Generally, the endocrine profile of smokers, including FSH, LH, estradiol, and testosterone is abnormal [44]. These data show that smoking disturbs the HPG axis. In opposite, there is a report that shows nicotine in humans increases estradiol secretion in granulosa cells [45]. Species differences, dose and length of treatment with nicotine, the difference in nicotine preparation, and the source of cells can be considered the reason for these conflicting results [9, 44–46].

FSH and LH stimulate antral follicles growth [47]. FSH by inducing estradiol production, which stimulates CAT production in the dominant follicles, prevents follicular apoptosis [48]**.** Our findings showed a reduced level of estradiol in the nicotine-treated group. In this regard, it has been shown that cigarette smoking affects steroidogenesis [44, 49]. In women smokers, urinary estradiol is lower than in non-smokers [50].

Ginseng as a phytoestrogen exerts its effect through binding to ERs (i.e. ERα or β) or through ERs’ activation and upregulation [27]. Ginseng extract increases cells growth via ERs [27]. Estradiol is a stimulator for the proliferation of granulosa cells. It is also important for follicle growth. It seems PG through estradiol elevation promotes ERs’ activation or upregulation, which results in cell proliferation [27].

An in vitro study about nicotine effects on granulosa cells showed the inhibitory effect in the highest dose in the bovine ovarian follicles [46]. Nicotine inhibits the production of androgens by theca interna cells which are isolated from bovine follicles. Following the inhibitory effects of nicotine on the production of androgen, the function of the follicles will be disturbed [46]. Similarly, treated mice with nicotine had a reduced level of estradiol [4]. Nicotine exerts its inhibitory effects on steroidogenesis by inhibiting aromatase that converts androgens to estrogens [51]. It is reported that androgen production in bovine ovarian follicles is inhibited by nicotine [46].

An insignificant reduction was observed in progesterone levels in the nicotine group of the present study. There are conflicting opinions about smoking’s effects on human granulosa-lutein cell function; therefore, this subject needs further investigations. Barbieri et al*.* in an in vitro study showed nicotine exposure leads to aromatase inhibition in granulosa cells [51]. Miceli et al. found a decrease in progesterone releasing, in the culture of human luteal cells, which were treated with nicotine or M-nicotine [52]. Weiss et al. showed no effect of nicotine and cotinine on estradiol and progesterone production in human granulosa cells [53]. Likely, it has previously been reported ginseng increases the progesterone and testosterone levels and alleviates reprotoxicity during pregnancy in rats under treatment with phthalate and bisphenol A [54].

Our findings indicated that the administration of nicotine during 30 days causes follicle loss. Similarly, Bordel et al. reported low growth of follicles [55], and Sezer et al. [13]. and Musanejad et al*. *[40] reported a low count of follicles under the influence of nicotine. Likely follicle count reduces in the ovary of smokers [56]. Our study indicated that primordial follicle numbers were not affected significantly in different groups. Similarly, Musanejad et al*.* showed no change in the number of primordial follicles in one-day-old mice pups after nicotine treatment. However, they showed a reduction in primordial follicles count in fifty-six-day-old mice [40]. Similar to our study, secondary and antral follicles were reduced in adult mice and rats, suggesting the demise of follicles during development [8, 9].

Contrary to the present study, the primordial follicle numbers were reduced in nicotine-treated rats or mice [13, 40, 55, 56]. These differences may arise from differences in species, animal age, duration of treatment, route of administration, and type of studies (in vivo or in vitro).

Our data revealed treatment with nicotine plus ginseng, especially in the dose of 1 g/kg increased total numbers of healthy follicles and reduced atretic follicles in the ovary. Zare et al*.* reported elevation of both numbers and size of primordial follicles in ovaries of three-month-old female rat infants born to diabetic mothers, which was treated with ginseng extract [22]. A recent study has reported the disappearance of ovarian cysts after a 14-day oral regimen of Korean red ginseng extract in an animal model [57]. Similarly, genistein, a phytoestrogen that binds to the ERs, protects ovarian follicles against radiotherapy and increases primordial, preantral, and antral follicles, and reduces atretic follicle counts through inhibition of apoptosis markers and upregulation of ERs in the ovary [58]. In a rat model of polycystic ovaries induced by estradiol valerate, ginseng normalizes ovarian morphology, reduces antral follicle counts, and increases corpus luteum numbers via regulation of nerve growth factor expression [59]. It is possible that ginseng through the HPG axis and alteration of sex hormones and therefore amelioration of ROS and apoptosis could alleviate the toxic effects of nicotine on folliculogenesis that our findings confirmed.

Reduction of antioxidant levels, both enzymatic and non-enzymatic forms affects women’s fertility [60]. Elevation of apoptotic markers in this study and also follicle loss may be due to the elevation of MDA activity in association with CAT and SOD activity reduction, which was observed in the nicotine-treated group. ROS has important physiological roles in women’s reproduction, including ovarian steroidogenesis, oocyte maturation, ovulation, blastocyst formation, fertilization, and embryo implantation. Any imbalance in pro-oxidants and antioxidants may damage these steps, and result in subfertility or infertility [60].

Likewise, a previous study has shown that treatment with nicotine increases the MDA level and reduces CAT and SOD activity in the ovary, liver, kidney, and pituitary tissues [61]. Additionally, smoke exposure in rats reduces tissue and serum glutathione peroxidase (GPx), and SOD and CAT activities [56]. Previous studies have shown that there is a link between oxidative stress and estrogen deficiency. Estrogen reduces oxidative stress through ROS scavenging. It is possible that in this study reduced level of estradiol in the nicotine group leads to MDA elevation and CAT and SOD activity reduction in serum or ovarian tissue [62, 63].

As a result of hormonal and follicular growth disruption, corpus luteum numbers as a marker of ovulation were reduced. This finding was similar to previous studies [8, 9, 17]. After ovulation, both FSH and LH stimulate corpus luteum formation from follicle remnants. The corpus luteum secretes progesterone, which is important for supporting the luteal phase and embryo endometrial implantation [42, 43]. An insignificant reduced level of progesterone in the nicotine group may be due to the lower numbers of corpus luteum, which was confirmed by our results.

Steroidogenesis is considered an important source of ROS production in the corpus luteum. However, there are high levels of antioxidant enzymes in the corpus luteum including catalase, SOD, and glutathione peroxidase, which protect luteal cells against oxygen radicals produced during steroidogenesis [64]. It has been reported that SOD protects the corpus luteum against superoxide radicals to stimulate the luteal cells for the production of progesterone [64]. During the regression of the corpus luteum, oxygen radicals are increased inside the corpus luteum, and the production of progesterone is inhibited by ROS [64, 65]. In confirmation of the above-mentioned data, our results indicated sex hormonal disturbances in the nicotine group along with elevation of MDA content and reduced activity of SOD in the ovary. We also showed an elevation in levels of FSH, LH, estradiol, and progesterone in the co-treated groups. These changes were associated with a reduction in MDA content of the ovary and a higher SOD activity in the ovary of co-treated mice.

Antioxidant activity of ginseng has been shown both in humans and experimental animals [63, 66]. Previous studies indicated downregulation of ROS by ginseng causes antioxidant activities [67]. Elevation of estradiol in co-treated groups with nicotine and ginseng may be another cause of MDA reduction in serum or ovary tissue of studied groups. Our findings showed an elevation of CAT and SOD activities in serum and ovary tissue in the co-treatment groups. Similar to our study, ginseng treatment in animal models increases CAT and SOD activities and reduces the MDA level in various tissues such as the heart, lung, liver, and kidney [26].

Another possible mechanism of ovarian follicle loss may be the process of apoptosis; in this regard, our findings showed a significant increase in the Cyt c and Bax expression and a decrease in the Bcl-2 expression in ovarian tissue in the nicotine group. Following ovarian toxicity and damages, apoptosis is induced. The intrinsic pathway in the apoptosis process is regulated by pro- and anti-apoptotic members of the Bcl-2 superfamily, which affect Cyt c release from mitochondria. During the apoptosis process, the mitochondrial function is impaired, leading to Cyt c release into the cytoplasm [68]. Cyt c is detected in the cytoplasm after high damages in both acute and chronic cell injuries [69]. Our data showed significant alterations in ovarian apoptotic and anti-apoptotic markers, following treatment with nicotine. It represents that the cytotoxic effect of nicotine on the ovary is in part done via the apoptotic pathway.

Likely, Sezer et al. showed an increased level of granulosa cell apoptosis in rat ovarian follicles, following treatment with nicotine [13] and increased apoptosis in rat granulosa cells, which were exposed to nicotine in their fetal or neonatal period [19]. For having normal follicle growth, inhibition of follicular atresia and granulosa cell apoptosis and coordination of ROS and enzymatic antioxidants are very important [48]. Ginseng has anti-apoptotic effects in a rat model of polycystic ovarian syndrome induced by dehydroepiandrosterone [28]. The anti-apoptotic properties of ginseng also have been shown in neuroblastoma cells via ER β-mediated phosphatidylinositol-3 kinase/Akt signaling [70].

The other probable mechanism for follicle loss in the nicotine group may be alteration in proliferative activity of the ovarian somatic cells. Our findings showed nicotine significantly reduced granulosa and theca cell proliferative indexes of pre-antral and antral follicles. In this regard, a previous study showed a reduction of granulosa cells in rat ovaries induced by nicotine treatment [13]. Fetal or neonatal exposure to nicotine also reduces proliferation and elevates granulosa cell apoptosis in rats [21]. In vitro studies on oocytes, have shown that nicotine blocks meiotic division in metaphase-I through disturbances of meiotic spindle and chromosome [16, 17].

We showed that co-treatment with nicotine plus ginseng elevated proliferative index in somatic cells. There are three kinds of somatic cells in the ovary including granulosa, theca, and somatic cells [42]. There is an interaction among the granulosa cells, stromal cells, and theca cells, and oocytes in the ovarian follicles and cell interactions are important in the development of follicles [71]. Ovarian stromal cells are located both in the cortex and medulla parts of the ovary. They are fibroblast-like cells that create the organizational scaffolding for the organ-specific cells. Theca cells are located in the periphery of the follicles and separated from granulosa cells by a basal membrane [71]. Theca layer consists of the inner theca, interna and outer theca, externa layers. Their roles are follicular and structural support, production of androgen for granulosa cells for the synthesis of estradiol, and stimulation of growth of early follicles by androgens. The granulosa and interna theca cells synthesize estradiol in pre-ovulatory follicles [41]. It is known that the critical point of the estradiol level leads to LH surge and, then ovulation occurs [42]. Theca cells also secret some factors to regulate the function of granulosa cells, including insulin-like growth factor, transforming growth factor-beta, and bone morphogenetic protein. Theca cells possess LH receptors and these cells undergo a “luteinization” phase and directly produce progesterone in the corpus luteum [40, 42, 71]. Disturbances in theca cells may result in ovary diseases such as ovarian cancer, premature ovarian failure, and polycystic ovary syndrome [72]. Granulosa cells possess FSH receptors and, it is essential for the development of the follicles from the primary stage to the pre-antral stage. There is cell–cell contact between oocyte and granulosa cells. Both proliferation and expression of apoptosis in granulosa cells are associated with ovarian stroma/theca cells. Both granulosa cells and stroma/theca cells have ER. Granulosa cells mostly have ERβ and stroma/theca cells mostly have ERα [73]. It means that granulosa and stroma/theca cells are an important target of estrogen, which is essential for cell survival stimulation. It is known that estrogen promotes folliculogenesis, increases gonadotropin receptor expression, and inhibits cell apoptosis and subsequent follicular atresia [42]. In this regard, a previous study has reported a reduction of ERα both in the uterus and oviduct in the nicotine-treated mice [4].

It has been shown that the ERα knockout mice (αERKO) had the most severe ovarian phenotype, their follicles did not develop and their ovulation failed and they had hemorrhagic cysts, which resulted in infertility [73]. Zhang et al*.* reported that the effect of ginsenosides and FSH on the promotion of oocytes’ meiotic maturation is synergistic [74]. Ginseng can stimulate ERs’ formation on granulosa cells, which leads to higher responsiveness to estradiol, FSH, and LH; therefore, granulosa cell survival and follicle numbers elevate. Ginseng also stimulates meiosis maturation in the oocyte [74]. Additionally, ginseng can up-regulate the expression of ERα and ERβ in reproductive tissues [28, 30]. It is possible that in this study ginseng in the co-treated groups have protected the ovaries against nicotine through modifying the ERs. However, we did not evaluate ERs and it suggested to be performed in future studies.

Improvement in the estradiol, progesterone, FSH, and LH levels in the co-treated groups may be due to a reduction of apoptotic and oxidative markers and an increase in the proliferative indexes of somatic cells that our findings confirmed. Due to there is interaction between the somatic cells and oocytes, therefore, proliferation or apoptosis of one of these cells may result in impaired follicle growth.

We used the Ki-67 marker to show proliferation, which is expressed in all active phases of the cell cycle. In our observation, proliferating granulosa cells were mostly observed in pre-antral and antral follicles. Likewise, the lack of PCNA expression in granulosa cells of the primordial follicles has been shown in a previous study [75]. Our findings did not show any changes in the proliferative index of granulosa cells in primordial and primary follicles, following treatment with nicotine. It is known that the growth of primordial follicles is not dependent on gonadotrophins, as FSH receptors are found on the follicles with one or two layers of granulosa cells. In vivo and in vitro studies have shown that FSH increases granulosa cell proliferation [76]. One of the mechanisms in the nicotine group, following estradiol reduction, is the reduction of FSH receptors and ER, which leads to a low rate of proliferation in small primary follicles. In this regard, it has been shown that treatment with nicotine in adult mice reduces ERα both in the endometrium and oviduct [4]. The other probable decrease in small follicle count in this study may be due to the direct toxicity effect of nicotine on oocyte and granulosa cells in small follicles [8]**.** A decrease in granulosa proliferation index is considered as a mark for the elevation rate of atresia, in pre-antral and antral follicles in the nicotine group [76]; this was consistent with our findings. In keeping with our findings, cigarette smoke exposure in young mice reduces granulosa cell proliferative index in antral follicles without any effects on primary and preantral follicles [77]. However, Sezer et al. showed proliferation index reduction in all types of follicles, even primordial and primary ones [13].

Similarly, ginseng can protect gonadal function in hypothyroid female rat models; maintaining FSH, LH, and testosterone in the normal range [78]. Ginseng also protects ovarian follicles in letrozole-induced polycystic ovarian syndrome [57].

Our work was an animal study. Although there are many similarities in physiology between mice and humans, they are not the same in whole aspects; this was one of our research limitations. The other limitation was the duration of the study, which lasted 30 days. However, human smokers, usually smoke for many years. Definitely, long courses of treatment may have different effects compared to short courses, and this may cause contradictions in some findings in different studies. We used nicotine that is one of the cigarette ingredients and therefore may not have the same effects of whole cigarette smoke. However, our animal study provides comprehensive short-term information about nicotine effects on ovaries, as well as the benefits of ginseng on female fertility parameters.

In this study, we treated mice with nicotine and ginseng. Therefore, we used a whole animal model. In these whole animal models, the metabolism of the chemical substances can be performed by the organs other than the ovary, such as the kidney and liver under the influence of the HPG axis. Additionally, these models prevent some artificial changes that may arise following in vitro culture, such as ROS elevation [79]. The exact mechanisms of ginseng and nicotine on follicle growth, cell proliferation, and apoptosis need further evaluation. 

In conclusion, this study showed that ginseng protects folliculogenesis via alteration of HPG axis, induction of proliferation in ovarian somatic cells, reduction of lipid peroxidation, and down-regulation of apoptotic markers in mouse ovary treated with nicotine.

## Data Availability

All data during this study are included in this published article.

## Abbreviations

PG: Panax ginseng
RT-PCR: Reverse transcription polymerase chain reaction
MDA: Malondialdehyde
CAT: Catalase
FSH: Follicle-stimulating hormone
E2: 17βEstradiol
ERs: Estrogen receptors
PR: Progesterone receptor
AR: Androgen receptor
PCNA: Proliferating cell nuclear antigen
Cyt c: Cytochrome c
BSA: Bovine serum albumin
HRP: Horseradish peroxidase
LH: Luteinizing hormone
